# Prognostic Value of Chest CT Volumetric Analysis in Patients with Malignant Pleural Mesothelioma

**DOI:** 10.3390/jcm14051547

**Published:** 2025-02-25

**Authors:** Elisa Baratella, Eleonora Ercolani, Antonio Segalotti, Marina Troian, Stefano Lovadina, Fabiola Giudici, Pierluca Minelli, Barbara Ruaro, Francesco Salton, Maria Assunta Cova

**Affiliations:** 1 Radiology Unit, Department of Medical Surgical and Health Sciences, University Hospital of Cattinara, 34149 Trieste, Italy; 2 Thoracic Surgery Unit, Cardiothoracic and Vascular Department, University Hospital of Cattinara, 34149 Trieste, Italystelova@hotmail.com (S.L.); 3 Cancer Epidemiology Unit, Centro di Riferimento Oncologico di Aviano (CRO), IRCCS, 33081 Aviano, Italy; fabiola.giudici@cro.it; 4 Pulmonology Unit, Department of Medical Surgical and Health Sciences, University of Trieste, 34149 Trieste, Italy; barbara.ruaro@yahoo.it (B.R.);

**Keywords:** volumetric analysis, chest CT, mesothelioma, prognosis

## Abstract

**Background/Objectives**: Malignant pleural mesothelioma (MPM) is a rare, aggressive cancer linked to asbestos exposure and with poor overall survival. In recent years, CT volumetric analysis has gained increasing interest as a more accurate method for assessing tumor burden. This study aims to evaluate the prognostic value of chest CT volumetric analysis in MPM, comparing tumor volume with tumor thickness measurements and survival outcomes. **Methods**: This is a retrospective, observational analysis of all patients undergoing diagnostic thoracoscopy between 2014 and 2021 at the University Hospital of Cattinara (Trieste, Italy). Inclusion criteria were as follows: age ≥ 18 years, histological diagnosis of MPM, and the availability of at least one contrast-enhanced chest CT scan at the time of diagnosis. For each patient, the tumor thickness was measured on the axial plane at three levels (upper, middle, and lower hemithorax). Tumor and effusion volumes were calculated with the RayStation^®^ software version 11.7.174 (HealthMyne^®^, Madison, WI, USA). **Results**: A total of 81 patients were eligible for analysis. Maximum and mean tumor thickness were strongly associated with survival, with higher thicknesses correlating with an increased risk of death (adjusted hazard ratio per doubling (aHR) of 1.97 (95%CI: 1.40–2.77) and of 2.23 (95%CI: 1.56–3.20), *p* < 0.001)), respectively, while the effect of the tumor volume on survival was nevertheless significant but less impactful (aHR = 1.26 (1.10–1.45, *p* < 0.001)). The presence and volume of effusion did not correlate with survival (*p* = 0.48 and *p* = 0.64, respectively). **Conclusions**: This study supports the role of quantitative parameters for staging MPM, particularly given the frequent discrepancies between clinical and pathological staging when relying solely on qualitative measures.

## 1. Introduction

Malignant pleural mesothelioma (MPM) is a rare and aggressive neoplasm originating from the pleura [1,2,3]. The risk of developing MPM is strongly correlated with the duration and intensity of asbestos exposure, and malignant evolution typically occurs several decades after initial exposure, often 20 to 50 years later [1,2,3,4]. Despite being banned in many countries, the continued mining and export of asbestos, particularly in developing countries, contributes to a rising global incidence, with most cases being diagnosed at an advanced stage, adding to poor overall survival [1,2,3,4,5].

MPM typically begins insidiously and progresses rapidly from the pleural membranes to nearby structures, such as the lungs, chest wall, and mediastinum. As the disease advances, it can spread to regional lymph nodes and, in later stages, to distant metastases, including the liver and bones. Without effective treatment, MPM usually leads to death within 12–18 months, although this timeline can vary depending on the timing of diagnosis and available therapies [1,2,4,6].

The diagnosis of MPM primarily relies on imaging modalities, particularly chest CT scans, which offer a detailed visualization of the pleura, mediastinal structures, and other thoracic organs [7,8,9]. While traditional staging systems, such as the TNM classification, provide valuable insight into tumor extent and nodal involvement [10,11,12,13], the challenge remains in assessing the tumor burden and treatment response quantitatively. This has led to the adoption of Response Evaluation Criteria in Solid Tumors (RECIST) criteria [14,15], which are often employed to monitor changes in tumor thickness during therapy. However, these measurements are typically two-dimensional, limiting their ability to capture the full complexity of tumor growth and its response to treatment.

In addition to CT, other diagnostic modalities such as MRI and PET-CT are also available for use in the staging and restaging of mesothelioma. The role of MRI in mesothelioma imaging has been widely discussed over the past 20 years. Due to its high spatial and contrast resolution, MRI has been shown to be superior to CT in detecting the loco-regional involvement of mesothelioma, making it a valuable tool for staging and surgical planning [16]. However, there are limitations to its use in clinical practice, including high costs, limited access to the exam, and the time required to acquire the images, especially in patients with respiratory difficulties [17]. As such, MRI is typically reserved for cases with suspected infiltration of an adjacent structure within the pleural space, such as the diaphragm, mediastinum, and chest wall [18].

PET-CT plays a significant role in the evaluation of patients with mesothelioma, both during staging—to identify occult metastases, especially extra-thoracic and lymph node metastases, which may not be recognized using CT—and during restaging [19]. Additionally, PET-CT allows for the evaluation of lesion uptake, which can serve as an indicator of aggressiveness, as demonstrated in other cancers [20]. In recent years, there has been a growing interest in volumetric analyses using chest CT as a more accurate method for quantifying tumor burden [3,4,5,6,7,8,9,13,14,15]. Volumetric CT offers distinct advantages over traditional methods by enabling the assessment of tumor volume in three dimensions, thus providing a more comprehensive evaluation of tumor size, distribution, and changes over time [21]. The potential for volumetric measurements to serve as a prognostic indicator for MPM has prompted several studies to explore its correlation with disease staging and patient outcomes. Specifically, the correlation between tumor volume, pleural effusion, and overall survival has emerged as an area of significant interest, suggesting that these parameters may offer insights into disease progression and treatment efficacy [22,23,24].

This study aims to evaluate the prognostic value of chest CT volumetric analyses in patients with MPM. By comparing the tumor volume with RECIST tumor thickness measurements and staging, we seek to investigate whether volumetric assessments can provide additional prognostic information beyond traditional imaging approaches. Moreover, we aim to explore the relationship between the extent of pleural effusion at diagnosis and patient survival, contributing to a more robust understanding of the disease’s behavior and response to treatment. Through this investigation, we hope to enhance the clinical utility of chest CT as a tool for better prognostication and tailored treatment strategies in MPM patients.

## 2. Materials and Methods

This is a retrospective, observational, monocentric study conducted on all patients undergoing diagnostic thoracoscopy between 2014 and 2021 at University Hospital of Cattinara (Trieste, Italy), approved by the Local Ethical Committee (CEUR-2022-Os-208) and carried out in accordance with the Declaration of Helsinki. Inclusion criteria for analysis were as follows: 18 years of age or older; diagnosis of MPM confirmed on histological examination; and availability of at least one contrast-enhanced chest CT-scan at the time of diagnosis.

**Figure 1:**
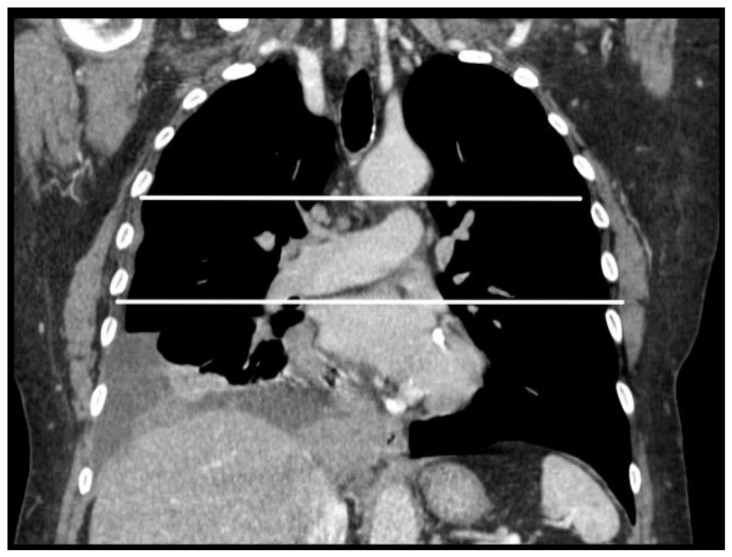
Coronal section showing the division into upper, middle, and lower hemithorax.

**Figure 2:**
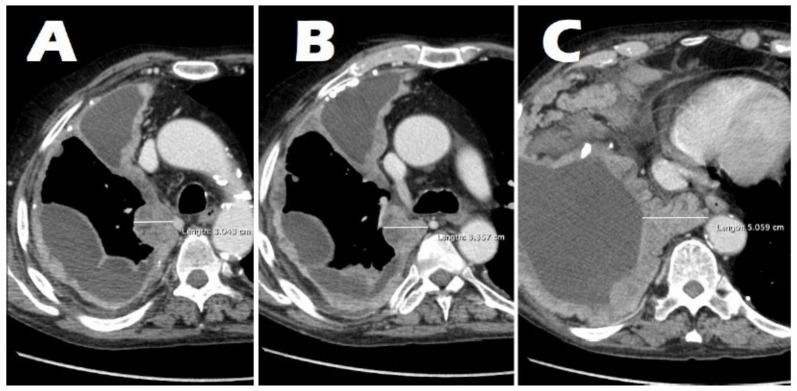
Tumor thickness measured perpendicularly at the level of the upper (**A**), middle (**B**), and lower (**C**) hemithorax.

For each patient, tumor thickness was measured in millimeters on the axial plane at three levels (Figure 1): upper hemithorax (i.e., from the apex of the lung to the lower margin of the aortic arch); middle hemithorax (i.e., between the lower margin of the aortic arch and the upper margin of the left atrium); and lower hemithorax (i.e., between the upper margin of the left atrium and the diaphragm). Measurements were taken perpendicularly to either the chest wall or the mediastinum, on the plane of greatest thickness of tumoral lesions, using the RayStation^®^ software version 11.7.174 (HealthMyne^®^) (Figure 2). In cases where no macroscopically evident pathological tissue was visible on CT images, an arbitrary value of 5 mm was assigned for the purposes of this analysis in accordance with the most recent literature [25].

Tumor thickness was measured in consensus by two radiologists with 3 and 4 years of clinical experience, respectively. Any discrepancy was resolved by a third radiologist with 15 years of experience in thoracic imaging. Both the maximum thickness (i.e., the highest value of the three measurements) and the average thickness (i.e., the mean value among the three measurements) were considered for analysis.

**Figure 3:**
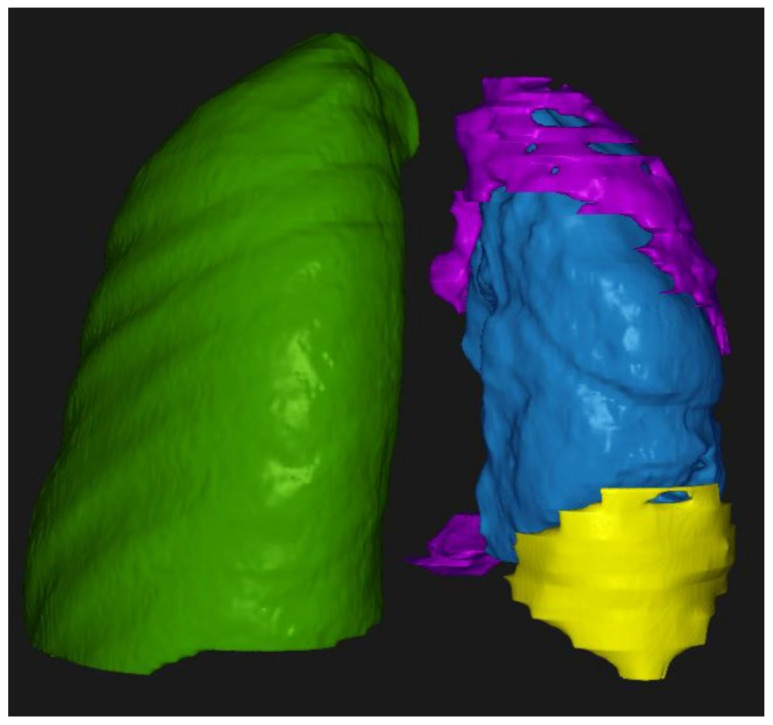
Three-dimensional representation of volumes calculated with RayStation^®^. In green, the right lung is shown. In blue, the left lung is shown. In purple, the tumor volume is shown. In yellow, the pleural effusion is shown.

The RayStation^®^ software (HealthMyne^®^), commonly employed by radiation oncologists for treatment planning, was used to calculate both tumor and effusion volumes (Figure 3). The smart brush and smart interpolation tools were applied to segment and measure the volume of the tumor, starting from the planes where tumor thickness was measured with the RayStation^®^ software version 11.7.174 (HealthMyne^®^) and then proceeding axially. In the presence of pleural effusion, the spline tool was applied to segment and calculate the volume of the effusion, starting from the bases and proceeding upwards, along the axial plane. For the purposes of this study, in case of minimal or nodular growth patterns, the three largest lesions were included in the analysis, omitting the smaller ones, whereas in case of circumferential disease, the analysis evaluated the lesion in its entirety.

### 2.1. CT Acquisition Protocol

All CT scans were performed using a 256-slice multi-detector CT system (Brilliance iCT 256, Philips Healthcare, Best, The Netherlands). The patients were invited to hold their breath with tidal inspiration during scanning in the supine position. Technical parameters were as follows: 128 × 0.625 mm section collimation; 0.27 s rotation time; 0.99 normalized pitch; 160 mm z-axis coverage; 0.3 mm reconstruction interval; 5 mm reconstruction thickness; 120 kV tube voltage; 280–400 mA effective tube current (depending on the patient’s size); field of view variable in relation to the patient’s body. CT images were analyzed at standardize mediastinal window settings (i.e., window level 400–500 HU, window width 20–40 HU).

### 2.2. Staging

The 8th Edition of the TNM staging for MPM provides a structured approach to assess the extent of tumor spread, although it does not account for factors such as histologic subtype, molecular characteristics, and patient’s overall status [12,14]. The T category evaluates the size and extent of the primary tumor, ranging from localized disease confined to the pleura to more advanced stages where the tumor invades nearby structures like the diaphragm or mediastinum. The N category reflects the spread of the tumor to regional lymph nodes, with higher stages indicating more extensive lymph node involvement, including contralateral or distant nodes. Finally, the M category assesses the presence of distant metastases, which significantly impact the prognosis. Together, these categories classify MPM into stages, providing a standardized framework that facilitates clinical decision-making and helps predict outcomes and tailor treatments. The TNM classification and staging for MPM are reported in Table 1.

### 2.3. Statistical Analysis

Results were reported as mean ± standard deviation (SD) or median and interquartile range for continuous variables, and as absolute frequencies and percentages for categorical variables. The Shapiro–Wilk test was applied to continuous variables to verify normal distribution.

The Kruskal–Wallis test was used to evaluate possible associations between the several quantitative parameters such as the sum of thicknesses, the mean, the volumes, and the T parameter (*p*-value obtained from post hoc pairwise comparisons were adjusted with the Holm method). The Spearman correlation test was used to evaluate the correlation between percent pathological aerated volume and thicknesses (maximum and mean).

Overall survival was defined as the time from the date of diagnosis to death. For the purposes of this study, the date of the last follow-up was 15 March 2022: patients alive on that date were censored. The median follow-up was calculated using the Schemper method. Kaplan–Meier curves were used to visualize the survival distributions, and the Log-Rank test was performed to evaluate the differences between the groups. Since there are no validated cut-offs in the literature that evaluate the prognostic role of maximum thickness and mean thickness, a recursive partitioning analysis was used to detect optimal cut-offs that identify patients with a better/worse prognosis for survival (package “party” [R 4.0.2]).

Multivariable Cox models were performed to evaluate the impact of CT volumetric variables on survival, quantified with hazard ratios (HRs) and their 95% confidence intervals. Average tumor thickness, maximum tumor thickness, and tumor volume had right-skew distributions and, when analyzed as continuous variables, were log-transformed to the base 2; this allows for the interpretation of hazard ratios for log tumor volume to be interpreted as the increase in hazard per doubling in tumor volume values (the same interpretation for the other variables).

R software (version 4.0.2—R Foundation for Statistical Computing) was used for statistical analysis. Statistical significance was expressed as *p* < 0.05.

## 3. Results

Between 2014 and 2021, a total of 400 patients underwent diagnostic thoracoscopy at the University Hospital of Cattinara (Trieste, Italy). Of these, 134 patients were diagnosed with MPM, and 81 patients met the selection criteria to be included in this study. All characteristics of the study population are summarized in Table 2.

### 3.1. Patients’ Characteristics

Overall, there were seventy-three (90.1%) men and eight (9.9%) women, with a median age at the time of diagnosis of 73 (range 69–78) years. Exposure to asbestos was confirmed for sixty-seven (82.7%) patients, whereas exposure was uncertain for ten (12.3%) patients and virtually absent for four (4.9%) patients. At the time of last follow-up, 95.1% (*n* = 77) of patients were deceased.

### 3.2. Radiological Findings on Chest CT

The neoplasm was observed on the right side in 40 (49.4%) patients and on the left side in 41 (50.6%) patients. The tumor growth pattern was categorized as nodular in 42 (51.9%) patients, minimal in 28 (34.6%) patients, and circumferential in 11 (13.6%) patients. The mean average thickness (i.e., the average value of three measurements) was 12.4 mm [7.9–17.9] and the mean maximum thickness (i.e., the largest value of three measurements) was 18.3 mm [10.8–26.3]. The median volume of the tumor on CT scan was 27.7 mL [15.7–88.1] mL.

Overall, 70 (86.4%) of patients presented a pleural effusion at the time of diagnosis, with a median volume on the CT scan of 1784.13 (range 791.29–2705.10) mL.

### 3.3. Correlation Between Tumor Thickness and the T Parameter

Significant differences were found in the correlation between the mean tumor thickness and the T parameter (*p* = 0.001), particularly when comparing T1 vs. T4 tumors (median 7.5 mm vs. 19.8 mm, *p* < 0.001). Significant differences were also found in the correlation between the T parameter and maximum tumor thickness (*p* < 0.001), especially when comparing T1 vs. T4 (median 8.5 mm vs. 27.4 mm, *p* < 0.001) and T2 vs. T4 (median 12.6 mm vs. 27.4 mm, *p* = 0.031).

### 3.4. Correlation of Tumor Volume with Tumor Thickness and the T Parameter

**Figure 4:**
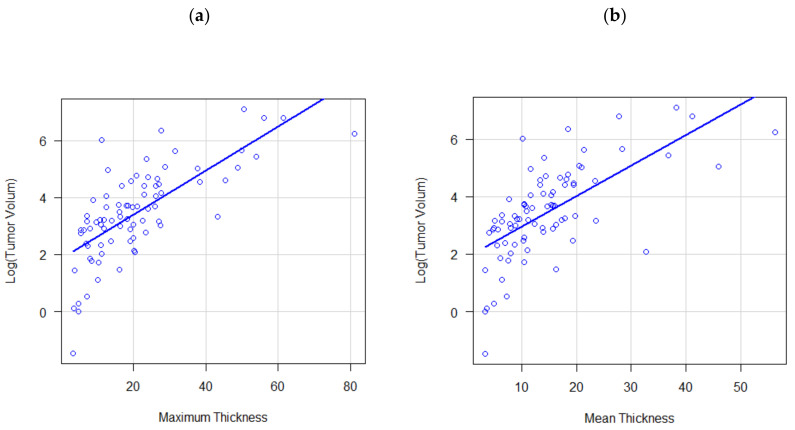
Correlation of tumor volume with mean tumor thickness (**a**) and maximum tumor thickness (**b**).

The tumor volume was significantly correlated with both the mean thickness (r = 0.69, *p* < 0.001) and maximum thickness (r = 0.74, *p* < 0.001) (Figure 4). Significant differences were also found in the correlation between the T parameter and tumor volume (*p* < 0.001), observing higher tumor volume values in T4 tumors with respect to all other T categories: T1 vs. T4 (median 11.7 mL vs. 124.5 mL, *p* < 0.001), T2 vs. T4 (median 18.4 mL vs. 124.5 mL, *p* = 0.004), and T3 vs. T4 (median 24.2 mL, vs. 124.5 mL, *p* = 0.014)

### 3.5. Survival Analysis

The median survival time for the 81 eligible patients (of whom 77 had died) was 13.3 months (95%CI: 10.2–17.6)

**Figure 5:**
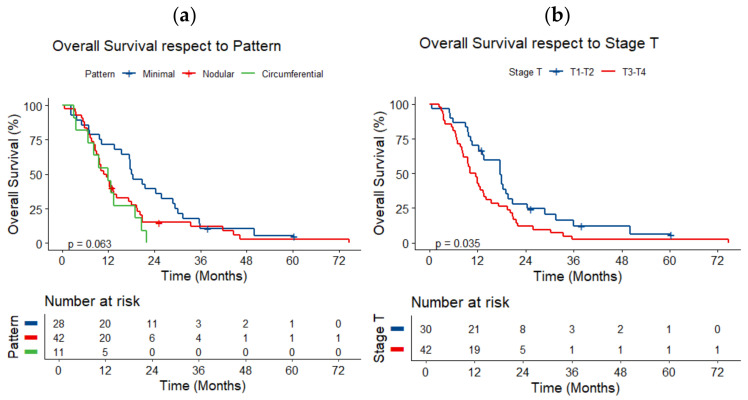
Overall survival respect to (**a**) tumor growth pattern and (**b**) stage T.

Patients with nodular or circumferential patterns had a worse prognosis than those with a minimal disease pattern (*p* = 0.063, Figure 5a). Significant differences were noted when comparing the minimal pattern with the circumferential pattern (median OS, respectively, 18.1 months vs. 11.9 months, adjusted *p*-value = 0.029). Lower survival was observed in patients in T3–4 stages compared to T1-T2 stages (median OS, respectively, 10.9 months vs. 17.7 months, *p* = 0.035, Figure 5b).

The maximum and mean tumor thickness were strongly associated with survival, where a higher thickness was correlated with increased risk of death (Table 3).

The univariable analysis of the effect of the log maximum and mean tumor thickness on survival indicated a 55% increase in risk of death per doubling of these variables’ values (HR = 1.55; 95%CI, 1.22–1.96 and HR = 1.54 (1.21–1.95), *p* < 0.001, respectively). Also, the tumor volume was correlated with survival but with a lower impact, showing a 19% increase in risk of death per doubling of volume (HR = 1.19; 95%CI, 1.08–1.32, *p* < 0.001). After adjusting for age, gender, T stage, histologic subtype, and treatment, the maximum tumor thickness, mean tumor thickness, and tumor volume were confirmed as prognostic factors (HR = 1.97 (1.40–2.77), HR = 2.23 (1.56–3.20), and HR = 1.26 (1.0–1.45)), respectively, Table 3).

**Figure 6:**
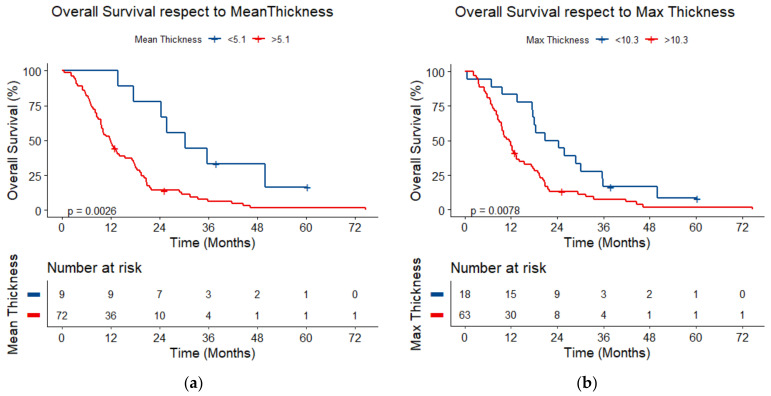
Overall survival with respect to (**a**) mean tumor thickness and (**b**) maximum tumor thickness.

When exploring the possibility of calculating a cut-off in order to identify patients with a worse prognosis, the optimal cut-off was found to be 5.1 mm for mean thickness, 10.3 mm for maximum thickness, and 4.3 mL for tumor volume, respectively. For a mean thickness above 5.1 mm, the median survival was 11.95 (95%CI: 9.65–14.18) months vs. 30.21 (95%CI: 13.69–50.01) months below this threshold (*p* = 0.003). For a maximum thickness above 10.3 mm, the median survival was 11.67 (95%CI: 9.55–13.45) months vs. 22.68 (95%CI: 17.55–30.21) months below this threshold (*p* = 0.008) (Figure 6).

**Figure 7:**
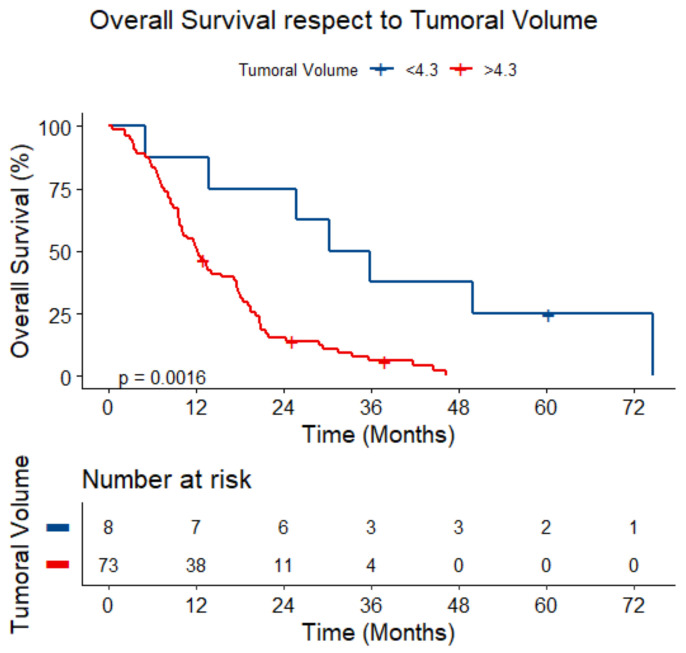
Overall survival respect to tumoral volume.

Lower volumes (<4.3 mL) presented a median survival of 33.07 (range 5.12–NA) months, whereas higher volumes presented a median survival of 12.23 (range 9.71–17.29) months (*p* = 0.002) (Figure 7).

**Figure 8:**
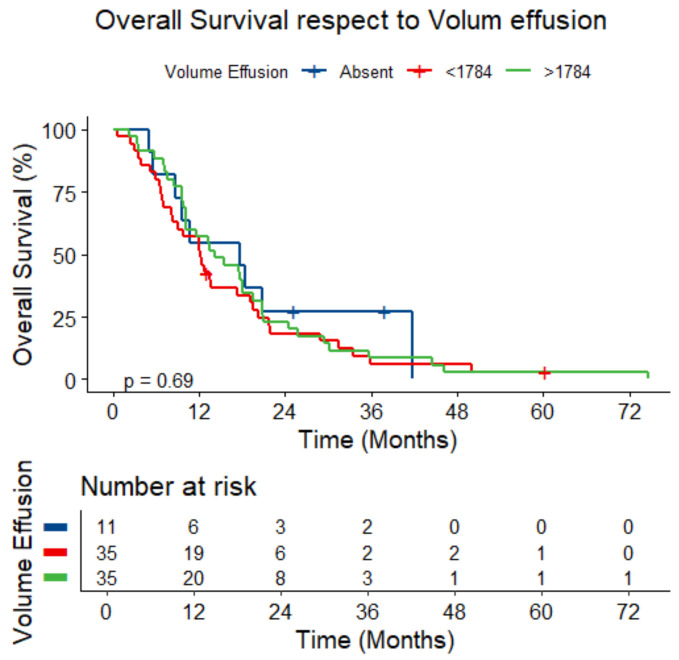
Overall survival respect to volume of pleural effusion.

The presence and volume of effusion did not correlate with survival (*p* = 0.478 and *p* = 0.636, respectively) (Figure 8).

## 4. Discussion

MPM is a rare cancer associated with asbestos exposure, characterized by an extremely poor prognosis. Life expectancy at the time of diagnosis is severely limited due to its aggressiveness and the scarcity of effective therapeutic options. To reduce the incidence of this neoplasm, most developed countries banned the use of asbestos between the 1980s and 1990s, although these measures have yet to result in a reduction of cases [1,2,3,4,5].

The TNM classification considers the pleura as a hollow organ, focusing not on the tumor’s size, as is typical with parenchymal organs, but on the extent of infiltration of adjacent structures [12,13,14]. Indeed, the development of MPM occurs along the pleura, often forming a “cage” around the lung, and is frequently accompanied by pleural effusion.

The clinical staging of MPM presents significant challenges, primarily due to the difficulty in accurately assessing the degree of infiltration of adjacent organs preoperatively, with CT scans showing an accuracy of only 46–55% [26]. Furthermore, the criteria used to detect lymph node metastases via CT yield a sensitivity of about 60% and a specificity of about 70% [8]. The limitations of these methods often lead to more advanced tumors being identified during surgical exploration than anticipated.

Our study’s findings on the importance of tumor thickness in determining the T parameter align with the existing literature data, particularly a study by the International Association for the Study of Lung Cancer (IASLC) and the International Mesothelioma Interest Group. In an analysis of 1987 patients, the role of the tumor thickness measured by CT in predicting survival and T staging was emphasized [27]. The present study demonstrated that a tumor thickness higher than 5.1 mm carried a significantly worse prognosis (median survival of 17.7 months vs. 24.2 months, *p* = 0.003).

Additionally, an IASLC study on 472 MPM patients found that clinical TNM staging matched pathological staging in only 36.2% of cases, with a significant risk of understaging (44.2%) and of modest overstaging (19.4%). According to this study, staging based on quantitative factors, such as three-dimensional tumor volume reconstructed via CT and maximum tumor thickness, would provide more accurate prognostic information than standard clinical TNM staging [21].

Currently, dimensional criteria are used to assess treatment response, with the RECIST guidelines focusing on three perpendicular tumor measurements across three axial sections. The sum of these measurements forms the “tumor diameter”, which is then used for comparison during follow-up [14,15,24]. While the use of quantitative values in RECIST is expected, as it enables comparisons between pre-treatment and post-treatment, the observed link between tumor thickness and overall survival indicates that incorporating these quantitative parameters into initial staging could provide a more accurate prognosis. This approach could give patients a clearer understanding of their disease’s progression.

Our study demonstrated a strong correlation between tumor thickness (specifically, mean tumor thickness and maximum tumor thickness) and the T parameter, as well as between tumor thickness and overall survival. Based on our analysis, the optimal cut-off values for prognosis prediction were a mean tumor thickness of 5.1 mm and a maximum tumor thickness of 10.3 mm. Patients with tumor thicknesses exceeding these values showed significantly reduced survival. Regarding tumor volumetric assessment, our study did not find it as effective in predicting outcomes, though it suggested a cut-off of 4.3 mL below which patients’ survival declined. However, these findings require validation in a larger sample population.

During the segmentation process, particularly with RayStation^®^ software version 11.7.174 (HealthMyne^®^), we encountered challenges in distinguishing tumor tissue from adjacent tissues due to a low contrast resolution. In particular, it was often difficult to differentiate tumor tissue from areas of lung atelectasis and, sometimes, the tumor infiltration of neighboring structures made it almost impossible to distinguish it clearly as the mass formed by tumor tissue and healthy lung parenchyma appeared relatively uniform.

Volumetric evaluation requires considerable effort and the measurement of tumor and pleural effusion volumes takes an average of 15–30 min per patient. However, volumetry offers the significant advantage of reducing interobserver variability. In the coming years, advancements in technology are expected to enable automated evaluations, thus eliminating the time-consuming process for operators and allowing volumetry to become a regular part of clinical practice. Currently, many software programs offer semi-automatic tools for simple operations, such as total aerated lung volume assessment or parenchymal density analysis (e.g., identifying healthy tissue from ground-glass opacities). If computational progress leads to semi-automatic tumor volume assessments, it could revolutionize both clinical practice and research.

A recent study published by a team from Glasgow [24] tested an artificial intelligence system based on a convolutional neural network, which learned autonomously from segmentation examples and successfully segmented volumes from other CT scans, yielding promising preliminary results despite occasional errors. This development suggests promising prospects for the integration of deep learning technologies.

Finally, the lack of correlation between pleural effusion at diagnosis and survival represents an interesting result. A meta-analysis of pleural effusions in cancer patients found it to be a negative factor for many cancers (i.e., HR 1.72, 95%CI 0.79–3.71), excluding mesothelioma (although only three small studies were available for meta-analysis) [28]. Considering that pleural effusion is most commonly found in pleural mesotheliomas with epithelial histology, which typically have a better prognosis than other subtypes, it might be expected to have a positive prognostic significance. However, our study failed to show a prognostic correlation for pleural effusion.

The present study has several limitations. Its retrospective design may affect the generalization and robustness of our findings. Additionally, the small sample size may limit the statistical power of our analysis, making it harder to detect subtle associations or to draw definitive conclusions. To address these limitations, future research with larger, well-powered sample sizes and prospective designs is crucial. Prospective studies would allow for better control over variables and minimize biases, ultimately helping to validate and strengthen the preliminary findings of our study.

## 5. Conclusions

Based on our study and the existing literature, an association between tumor thickness in MPM and its prognosis has emerged. Regarding tumor volume, our study suggests a potential correlation with prognosis, although we were unable to demonstrate it clearly. Furthermore, our study strongly rejects the hypothesis that survival is related to the volume of pleural effusion.

In light of our findings, we conclude that the use of quantitative parameters for staging in MPM should be seriously considered, particularly given the frequent discrepancies between clinical and pathological staging observed with the use of solely qualitative parameters.

## Figures and Tables

**Table 1:** Classification of pleural mesothelioma according to the 8th Edition of the TNM staging for MPM [12,14].

T1	Tumor involving the ipsilateral parietal or visceral pleura only.		
T2	Tumor involving ipsilateral pleura (parietal or visceral pleura) with invasion involving at least one of the following: diaphragmatic muscle; pulmonary parenchyma.		
T3	Tumor involving ipsilateral pleura (parietal or visceral pleura) with invasion involving at least one of the following: endothoracic fascia; mediastinal fat; chest wall, with or without associated rib destruction (solitary, resectable); pericardium (non-transmural invasion).		
T4	Tumor involving ipsilateral pleura (parietal or visceral pleura) with invasion involving at least one of the following: chest wall, with or without associated rib destruction (diffuse or multifocal, unresectable); peritoneum (via direct transdiaphragmatic extension); contralateral pleura; mediastinal organs (esophagus, trachea, heart, great vessels); vertebrae, neuroforamen, spinal cord, or brachial plexus; pericardium (transmural invasion with or without pericardial effusion).		
NX	Regional lymph nodes cannot be assessed.		
N0	No regional lymph node metastases.		
N1	Metastases to ipsilateral intrathoracic lymph nodes (including ipsilateral bronchopulmonary, hilar, subcarinal, paratracheal, aortopulmonary, paraesophageal, peridiaphragmatic, pericardial, intercostals, and internal mammary nodes).		
N2	Metastases to contralateral intrathoracic lymph nodes, metastases to ipsilateral or contralateral supraclavicular lymph nodes.		
M0	No distant metastasis.		
M1	Distant metastases present.		
Stage I A	T1	N0	M0
Stage I B	T2, T3	N0	M0
Stage II	T1, T2	N1	M0
Stage III A	T3	N1	M0
Stage III B	T1, T2, T3	N2	M0
	T4	Any N	M0
Stage IV	Any T	Any N	M1

**Table 2:** Characteristics of the study population.

Covariate	Total (*n* = 81)
Age (median [range]), years	73 [69–78]
Sex, n (%)	
Male	73 (90.1%)
Female	8 (9.9%)
Asbestos exposure, *n* (%)	
Yes	67 (82.7%)
No	4 (4.9%)
Uncertain	10 (12.3%)
Tobacco use, *n* (%)	
Yes	30 (37.1%)
No	15 (18.5%)
N/A	36 (44.4%)
Diabetes, *n* (%)	
Yes	21 (25.9%)
No	59 (72.8%)
N/A	1 (1.2%)
Arterial hypertension, *n* (%)	
Yes	48 (51.9%)
No	32 (46.9%)
N/A	1 (1.2%)
Coronary artery disease, *n* (%)	
Yes	15 (18.5%)
No	65 (80.3%)
N/A	1 (1.2%)
Tumor side, *n* (%)	
Right side	40 (49.4%)
Left side	41 (50.6%)
Histologic subtype, *n* (%)	
Epithelioid	60 (74.1%)
Sarcomatoid	8 (9.9%)
Biphasic	12 (14.8%)
Desmoplastic	1 (1.2%)
Tumor growth pattern, *n* (%)	
Minimal	28 (34.6%)
Nodular	42 (51.9%)
Circumferential	11 (13.6%)
Pleural effusion, *n* (%)	
Yes	70 (86.4%)
No	11 (13.6%)
Treatment, *n* (%)	
None treatment	12 (14.8%)
Only CT	16 (19.8%)
P/D	8 (9.9%)
CT + RT	7 (8.6%)
P/D + Chemotherapy	22 (27.2%)
P/D + CT + RT	16 (19.8%)
T parameter, *n* (%)	
T1	19 (23.5%)
T2	11 (13.6%)
T3	26 (32.1%)
T4	16 (19.8)
N/A	9 (11.1)
Average tumor thickness (median [range]), mm	12.4 [7.9–17.9]
Maximum tumor thickness (median [range]), mm	18.3 [10.8–26.3]
Tumor volume (median [range]), mL	27.7 [15.7–88.1]
Pleural effusion volume (median [range]), mL	1784.1 [821.6–2697.0]
Follow-up (median [range]), months	31.6 [22.2–43.6]

Legend. N/A—not available; CT—chemotherapy; RT—radiotherapy; P/D—pleurectomy and decortication (P/D); mm—millimeters; mL—milliliters.

**Table 3:** Cox prognostic models for chest CT volumetric parameters.

	Univariable Model		Multivariable Model	
Variable ^a^	Hazard Ratio (95%CI)	*p*-Value	Hazard Ratio (95%CI) ^b^	*p*-Value
Maximum thickness (continuous)	1.55 (1.22–1.96)	<0.001	1.97 (1.40–2.77)	<0.001
Mean thickness (continuous)	1.54 (1.21–1.95)	<0.001	2.23 (1.56–3.20)	<0.001
Tumoral Volume (continuous)	1.19 (1.08–1.32)	<0.001	1.26 (1.10–1.45)	<0.001

^a^ Variables were log2 transformed. ^b^ Adjusted for age, sex, T parameter, histologic subtype, and treatment: the hazard ratio can be interpreted as the risk increase by doubling variables values.

## Data Availability

The data presented in this study are available upon reasonable request from the corresponding author.

## References

[1] Jain M., Crites M.K., Rich P., Bajantri B. (2024). Malignant pleural mesothelioma: A comprehensive review. J. Clin. Med..

[2] Hajj G.N.M., Cavarson C.H., Pinto C.A.L., Venturi G., Navarro J.R., Lima V.C.C. (2021). Malignant pleural mesothelioma: An update. J. Bras. Pneumol..

[3] Liu B., van Gerwen M., Bonassi S., Taioli E., International Association for the Study of Lung Cancer Mesothelioma Task Force (2017). Epidemiology of environmental exposure and malignant mesothelioma. J. Thorac. Oncol..

[4] Remon J., Hendriks L.E.L., Bironzo P. (2022). Malignant pleural mesothelioma: New guidelines make us stronger for defeating this disease. Ann. Oncol..

[5] Fazzo L., Minelli G., De Santis M., Ceccarelli E., Iavarone I., Zona A. (2023). The epidemiological surveillance of mesothelioma mortality in Italy as a tool for the prevention of asbestos exposure. Int. J. Environ. Res. Public Health.

[6] Finn R.S., Brims F.J.H., Gandhi A., Olsen N., Musk A.W., Maskell N.A., Lee Y.C.G. (2012). Postmortem findings of malignant pleural mesothelioma: A two-center study of 318 patients. Chest.

[7] Kindler H.L., Ismaila N., Armato S.G., Bueno R., Hesdorffer M., Jahan T., Jones C.M., Miettinen M., Pass H., Rimner A. (2018). Treatment of malignant pleural mesothelioma: American Society of Clinical Oncology Clinical Practice Guideline. J. Clin. Oncol..

[8] Nickell L.T., Lichtenberger J.P., Khorashadi L., Abbott G.F., Carter B.W. (2014). Multimodality imaging for characterization, classification, and staging of malignant pleural mesothelioma. RadioGraphics.

[9] Rossi G., Davoli F., Poletti V., Cavazza A., Lococo F. (2021). When the diagnosis of mesothelioma challenges textbooks and guidelines. J. Clin. Med..

[10] Scherpereel A., Opitz I., Berghmans T., Psallidas I., Glatzer M., Rigau D., Astoul P., Bölükbas S., Boyd J., Coolen J. (2020). ERS/ESTS/EACTS/ESTRO guidelines for the management of malignant pleural mesothelioma. Eur. Respir. J..

[11] Berzenji L., Van Schil P.E., Carp L. (2018). The eight TNM classification for malignant pleural mesothelioma. Transl. Lung Cancer Res..

[12] AIOM Linee Guida Mesotelioma Pleurico Edizione 2021. https://www.iss.it/documents/20126/8403839/LG-305-AIOM_Mesotelioma.

[13] Euhus C.J., Ripley R.T. (2020). The staging of malignant pleural mesothelioma. Thorac. Surg. Clin..

[14] Bonomi M., De Filippis C., Lopci E., Gianoncelli L., Rizzardi G., Cerchiaro E., Bortolotti L., Zanello A., Ceresoli G.L. (2017). Clinical staging of malignant pleural mesothelioma: Current perspectives. Lung Cancer.

[15] Armato S.G., Nowak A.K. (2018). Revised modified Response Evaluation Criteria in Solid Tumors for assessment of response in malignant pleural mesothelioma (Version 1.1). J. Thorac. Oncol..

[16] Barreto I., Franckenberg S., Frauenfelder T., Opitz I., Lauk O. (2024). Potential advantage of magnetic resonance imaging in detecting thoracic wall infiltration in pleural mesothelioma: A retrospective single-center analysis. JTCVS Open.

[17] Volpi F., D’amore C.A., Colligiani L., Milazzo A., Cavaliere S., De Liperi A., Neri E., Romei C. (2022). The use of chest magnetic resonance imaging in malignant pleural mesothelioma diagnosis. Diagnostics.

[18] Katz S.I., Straus C.M., Roshkovan L., Blyth K.G., Frauenfelder T., Gill R.R., Lalezari F., Erasmus J., Nowak A.K., Gerbaudo V.H. (2023). Considerations for imaging of malignant pleural mesothelioma: A consensus statement from the International Mesothelioma Interest Group. J. Thorac. Oncol..

[19] Lopci E., Castello A., Mansi L. (2022). FDG PET/CT for staging and restaging malignant mesothelioma. Semin. Nucl. Med..

[20] Nowak A.K., Francis R.J., Phillips M.J., Millward M.J., Van Der Schaaf A.A., Boucek J., Musk A.W., McCoy M.J., Segal A., Robins P. (2010). A novel prognostic model for malignant mesothelioma incorporating quantitative FDG-PET imaging with clinical parameters. Clin. Cancer Res..

[21] Gill R.R., Yeap B.Y., Bueno R., Richards W.G. (2018). Quantitative clinical staging for patients with malignant pleural mesothelioma. J. Natl. Cancer Inst..

[22] Armato S.G., Francis R.J., Katz S.I., Ak G., Opitz I., Gudmundsson E., Blyth K.G., Gupta A. (2019). Imaging in pleural mesothelioma: A review of the 14th International Conference of the International Mesothelioma Interest Group. Lung Cancer.

[23] Rusch V.W., Gill R., Mitchell A., Naidich D., Rice D.C., Pass H.I., Kindler H.L., Friedberg J., Ginsberg M., Erasmus J. (2016). Malignant mesothelioma volumetric CT study group. A multicenter study of volumetric computed tomography for staging malignant pleural mesothelioma. Ann. Thorac. Surg..

[24] Kidd A.C., Anderson O., Cowell G.W., Weir A.J., Voisey J.P., Evison M., Tsim S., A Goatman K., Blyth K.G. (2022). Fully automated volumetric measurement of malignant pleural mesothelioma by deep learning AI: Validation and comparison with modified RECIST response criteria. Thorax.

[25] Bellini A., Dell’amore A., Giraudo C., Modugno A., Bernardinello N., Terzi S., Zambello G., Pasello G., Zuin A., Rea F. (2021). Computed tomography and spirometry can predict unresectability in malignant pleural mesothelioma. J. Clin. Med..

[26] Entwisle J. (2004). The use of magnetic resonance imaging in malignant mesothelioma. Lung Cancer.

[27] Nowak A.K., Chansky K., Rice D.C., Pass H.I., Kindler H.L., Shemanski L., Billé A., Rintoul R.C., Batirel H.F., Thomas C.F. (2016). The IASLC Mesothelioma Staging Project: Proposals for revisions of the T descriptors in the forthcoming eight edition of the TNM classification for pleural mesothelioma. J. Thorac. Oncol..

[28] Yang Y., Du J., Wang Y.S., Kang H.Y., Zhai K., Shi H.Z. (2022). Prognostic impact of pleural effusion in patients with malignancy: A systematic review and meta-analysis. Clin. Transl. Sci..

